# A common neonicotinoid pesticide, thiamethoxam, impairs honey bee flight ability

**DOI:** 10.1038/s41598-017-01361-8

**Published:** 2017-04-26

**Authors:** Simone Tosi, Giovanni Burgio, James C. Nieh

**Affiliations:** 1 Alma Mater Studiorum, University of Bologna, Department of Agricultural Sciences, Viale Fanin 42, 40127 Bologna, Italy; 2 Council for Agricultural Research and Economics, Honey Bee and Silkworm Research Unit, Via di Saliceto 80, 40128 Bologna, Italy; 3 University of California, San Diego, Division of Biological Sciences, Section of Ecology, Behavior, and Evolution, 9500 Gilman Drive, MC0116, La Jolla, CA 92093-0116 San Diego, USA

## Abstract

Pesticides can pose environmental risks, and a common neonicotinoid pesticide, thiamethoxam, decreases homing success in honey bees. Neonicotinoids can alter bee navigation, but we present the first evidence that neonicotinoid exposure alone can impair the physical ability of bees to fly. We tested the effects of acute or chronic exposure to thiamethoxam on the flight ability of foragers in flight mills. Within 1 h of consuming a single sublethal dose (1.34 ng/bee), foragers showed excitation and significantly increased flight duration (+78%) and distance (+72%). Chronic exposure significantly decreased flight duration (−54%), distance (−56%), and average velocity (−7%) after either one or two days of continuous exposure that resulted in bees ingesting field-relevant thiamethoxam doses of 1.96–2.90 ng/bee/day. These results provide the first demonstration that acute or chronic exposure to a neonicotinoid alone can significantly alter bee flight. Such exposure may impair foraging and homing, which are vital to normal colony function and ecosystem services.

## Introduction

Pollinators play an important environmental role by providing essential ecosystem services^1^. In particular, the honey bee, *Apis mellifera* L., 1758, is an important global pollinator of crops and native plants^1^. The decline of managed honey bee colonies has therefore raised concern about ecological impacts, crop production, food security and human welfare^2^. Although beekeepers can multiply colonies to offset some of these losses, beekeeping is becoming increasingly difficult and expensive^2^. Multiple factors, including disease and pesticides, contribute to poor honey bee health^3^. Among pesticides, attention has focused on the neonicotinoids^4^, neurotoxic insecticides that are globally used on multiple crops^5^. Neonicotinoids are environmentally persistent and systemic: they can be found in the nectar, pollen, and guttation droplets that bees collect^6, 7^. Moreover, exposure to even low concentrations of neonicotinoids can harm bee health via synergistic interactions between multiple stressors^3, 7, 8^.

Neonicotinoids and their degradation products are agonists of insect nicotinic acetylcholine receptors^5^ and have a wide variety of neural effects^8, 9^. These compounds can therefore harm bee foraging^10–13^, homing^14–17^, locomotion^18, 19^, navigation^20^, and colony health^17^. Although neonicotinoids are partially restricted in Europe^21^, they are still commonly used worldwide^5, 22^, and thus their sublethal impacts deserve further study. We focused on thiamethoxam (TMX), a second generation neonicotinoid that is widely used^5^ and persistent^6^, and is thus frequently found in multiple environmental substrates such as nectar, pollen, guttation, water, and bee hives^6, 23–25^.

Henry *et al*.^16, 17^ demonstrated that TMX reduced forager return rates to the nest, raising the interesting possibility that TMX impairs navigation, flight ability, or both. Subsequently, researchers demonstrated that sublethal doses of three different neonicotinoids (clothianidin, imidacloprid and thiacloprid) could impair honey bee navigation^20^. TMX may similarly impair navigation^26^, but we tested the hypothesis that it reduces the physical ability of bees to fly. Flight is essential for colony fitness and health because bees fly to collect all of their food and water. Blanken *et al*.^27^ recently showed that bees exposed to *Varroa destructor* and imidacloprid over 13 weeks had a decreased ability to fly. Because there was no effect of imidacloprid alone on bee flight ability^27^ it was not clear if neonicotinoids alone can reduce bee flight ability. TMX can alter forager flight muscle temperature^28^, and the results of Henry *et al*.^16, 17^ suggested that TMX could impair honey bee flight: we therefore focused on TMX, using tethered bees flying on flight mills to test their physical ability to fly^29–33^, measuring flight distance, duration, and velocity in exposed and control bees.

## Results

### Acute exposure elicited excitation: increased flight duration and distance

Each bee flew twice (before and after treatment) in this experiment. Control bees (both phases) flew for 1390 ± 168 seconds (mean ± s.e.m., 23 min) and covered 2145 ± 294 m (2.1 km) at an average velocity of 1.49 ± 0.05 m/s (5.4 km/h) and maximum velocity of 1.78 ± 0.05 m/s (6.4 km/h).

**Figure 1:**
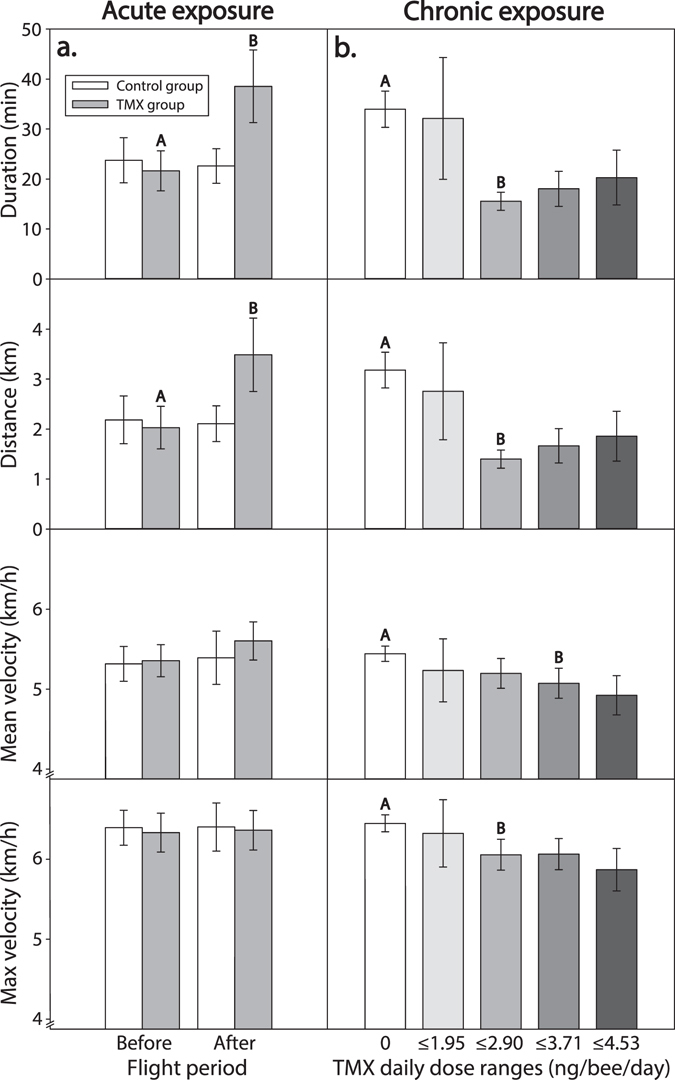
The effects of (**a**) acute or (**b**) chronic exposure to thiamethoxam (TMX) on forager flight ability. (**a**) In the acute experiment, we recorded flight duration, distance, mean velocity and maximum velocity before and after treatment; white bars are the control group (), grey bars are the TMX group (); the different letters indicate significant differences (LS Means contrast tests comparing before and after periods; *N*_*control group, before*_ = 16, *N*_*control group, after*_ = 16, *N*_*TMX group, before*_ = 23, *N*_*TMX group, after*_ = 23). (**b**) In the chronic experiment, we grouped the TMX daily doses (*N*_*TMX daily doses*_ = 46) in 5 TMX daily dose ranges (0, ≤1.95, ≤2.90, ≤3.71, ≤4.53 ng/bee/day). We pooled data from both days of exposure (1 or 2 days) because there was no significant effect of the number of days of exposure. Different shading reflects different daily dose ranges of TMX and different letters indicate significant differences (Least-Square Means contrast tests; *N*_*control*_ = 94, *N*_*32.5 ppb*_ = 44, *N*_*45 ppb*_ = 75). In the x-axis, we report the upper value of each bin range of TMX daily doses. Error bars show standard errors.

There was a significant effect of the interaction treatment × flight period on *duration* (*p* = 0.024, Fig. 1a, main effects in Table 1) because TMX-treated bees flew 78% longer after they consumed TMX (LS Means contrast test: *F*_1,37_ = 10.91, *p* = 0.002, Fig. 1a). As expected, control bees flew for similar durations in both phases (contrast test: *F*_1,37_ = 0.10, *p* = 0.75).

**Table 1:** Summary of the statistical results of the acute and chronic experiments.

TMX exposure	Flight parameter	Model fit (R^2^)	Colony effect (%)	Tested variable	DF numerator	DF denominator	*F* Ratio	*P-*Value
Acute	Duration	0.57	25	TMX treatment	1	34	0.99	0.326
				Flight period	1	37	3.38	0.074
				TMX treatment * Flight period	1	37	5.43	0.025
	Distance	0.57	25	TMX treatment	1	34	0.86	0.360
				Flight period	1	37	3.50	0.069
				TMX treatment * Flight period	1	37	5.57	0.024
	Mean velocity	0.44	4	TMX treatment	1	32	0.23	0.635
				Flight period	1	37	0.58	0.451
				TMX treatment * Flight period	1	37	0.16	0.693
	Max velocity	0.44	20	TMX treatment	1	32	0.008	0.928
				Flight period	1	37	0.008	0.929
				TMX treatment * Flight period	1	37	0.003	0.959
Chronic	Duration	0.13	2	TMX daily dose	1	210	18.30	<0.0001
				Days of exposure	1	25	3.72	0.065
	Distance	0.15	4	TMX daily dose	1	209	20.32	<0.0001
				Days of exposure	1	29	3.03	0.092
	Mean velocity	0.17	16	TMX daily dose	1	201	9.52	0.002
				Days of exposure	1	56	1.00	0.322
	Max velocity	0.18	16	TMX daily dose	1	201	9.90	0.002
				Days of exposure	1	56	1.35	0.250

REML variance component estimates of colony effects are reported as percentages (acute exposure: repeated-measures ANOVA_REML_; chronic exposure: Mixed Model_REML_, based on N_*TMX daily doses*_ = 46). *N*_*control group, before*_ = 16, *N*_*control group, after*_ = 16, *N*_*TMX group, before*_ = 23, *N*_*TMX group, after*_ = 23; *N*_*control*_ = 94, *N*_*32.5 ppb*_ = 44, *N*_*45 ppb*_ = 75.

Similarly, there was a significant effect of the interaction treatment × flight period on *distance* (*p* = 0.025, Fig. 1a, Table 1). Control bees flew similar distances in the before and after phases (contrast test: *F*_1,37_ = 0.10, *p* = 0.75), but treated bees flew 72% farther after they consumed TMX (contrast test: *F*_1,37_ = 10.59, *p* = 0.002).

There were no significant effects of treatment, flight period or their interaction on *mean velocity* or *maximum velocity* (p > 0.45, Fig. 1a, Table 1).

### Chronic exposure to TMX reduced flight ability

In this experiment, each bee flew only once after either one or two days of chronic exposure to TMX. The number of days of exposure had no significant effects on flight (p ≥ 0.07, Table 1). Control bees flew for 2036 ± 218 seconds (34 min) and travelled 3178 ± 357 m (3.2 km) with an average velocity of 1.51 ± 0.03 m/s (5.4 km/h) and a maximum velocity of 1.79 ± 0.03 m/s (6.4 km/h). The daily doses of TMX ingested significantly reduced flight ability for each flight parameter (*p* < 0.002, Table 1).

The TMX daily dose ingested significantly decreased flight *duration* (*p* < 0.0001, Table 1). For each 1 ng of TMX ingested daily by a forager, flight duration decreased by 20% (Mixed Model_REML_ estimate, based on *N*_*TMX daily doses*_ = 46). When we grouped bees by the actual TMX daily dose consumed (Fig. 1b), the foragers that ingested 1.96–2.90 ng/bee/day spent significantly less time flying than control bees (−54%, contrast test: *F*_1,169_ = 4.82, *p* = 0.029).

The TMX daily dose intake significantly reduced the total flight *distance* (*p* < 0.0001, Table 1). For each 1 ng of TMX ingested daily by the forager, their flight distance decreased by 23% (Mixed Model_REML_ estimate, based on *N*_*TMX daily doses*_ = 46). When we grouped bees by the TMX daily dose actually consumed (Fig. 1b), those that ingested 1.96–2.90 ng/bee/day flew significantly shorter distances compared to control (−56%, contrast test: *F*_1,207_ = 1.10, *p* = 0.019).

The daily dose of TMX ingested significantly reduced the *mean velocity* of the flights (*p* = 0.002, Table 1). We estimated that foragers flew 0.15 km/h slower for each 1 ng of TMX ingested daily (Mixed Model_REML_ estimate, based on *N*_*TMX daily doses*_ = 46). When we grouped bees by the TMX daily dose actually consumed (Fig. 1b), doses from 2.90–3.71 ng/bee/day significantly reduced *mean velocity* as compared to control (−7%, contrast test: *F*_1,202_ = 4.43, *p* = 0.037).

TMX significantly reduced flight *maximum velocity* (*p* = 0.002, Table 1). For each 1 ng of TMX ingested daily by the forager, their flight maximum velocity decreased by 0.15 km/h (Mixed Model_REML_ estimate, based on *N*_*TMX daily doses*_ = 46). When we grouped bees by the TMX daily dose consumed (Fig. 1b), doses from 1.96–2.90 ng/bee/day significantly reduced *maximum velocity* as compared to control (−6%, contrast test: *F*_1,197_ = 5.00, *p* = 0.026).

Daily consumption of the higher TMX sucrose solution was significantly higher than consumption of pure sucrose solution (+7%; control = 73 ± 15 mg/bee/day; 32.5 ppb = 75 ± 16 mg/bee/day; 45 ppb = 78 ± 14 mg/bee/day; Kruskal-Wallis Rank Sums, χ^2^ = 7.40, *p* = 0.02; Wilcoxon paired-sample test﻿, 0 versus 45 ppb: *Z* = 2.66, *p* = 0.008).

There were no significant effects of the interaction TMX daily doses × days of exposure (*p* > 0.11). There was no significant effect of TMX treatment (Fisher exact test, *p* = 0.065) on the number of bees that did not fly (23% over all treatments).

## Discussion

We present the first results demonstrating that sublethal acute or chronic neonicotinoid exposure is sufficient to significantly alter honey bee flight ability — affecting flight distance, flight duration, and flight velocity. Essentially, TMX had an excitatory short-term effect and a depressive longer-term effect. In the acute experiment, foragers consumed a single sublethal dose of 1.34 ng and subsequently increased their mean flight duration and flight distance by 78% and 72% in comparison with control bees, respectively (Fig. 1a). However, this increase in flight duration and distance is likely not beneficial because, at similar doses, TMX and other neonicotinoids cause flight disorientation^20, 26, 34^. Bees that fly more erratically for greater distances may thereby decrease their probability of returning home. This decline in the proportion of TMX-treated bees returning to the nest has been demonstrated, at the colony level, in two experiments by Henry *et al*.^16, 17^. In a similar study, Thompson *et al*.^35^ found no significant effect of TMX on honey bee homing ability. However, the study of Thompson *et al*.^35^ was carried out at a smaller spatial and temporal scale (i.e. *ca*. 1–2 field exposure units, with a single 2-ha treated field as compared to 63 field exposure units, with a total of 288-ha treated fields in Henry *et al*.^17^).

Chronic exposure is possible because foragers that consume a single sublethal dose of TMX can survive^16^ and return to forage at the same contaminated food sources. In fact, recent evidence shows that honey bees prefer sucrose solutions containing TMX or other neonicotinoids over pure sucrose^36^. Bees may therefore increase their consumption of contaminated food, exacerbating pesticide exposure. In our chronic experiment, bees drank significantly more sucrose solution when it contained TMX (+7%). We found that chronic exposure, which led to daily intakes of 1.96–2.90 ng TMX/bee/day, significantly decreased flight duration, distance, and velocity (Fig. 1b). TMX had the same significant negative effects on bees after one or two days of exposure (Table 1). Thus, chronic exposure to field-relevant daily doses of TMX (<2.94 ng/bee daily) over a single day was sufficient to impair bee flight ability.

Honey bees can forage up to 13.5 km from the colony, depending on forage availability and quality, and colony health^37^. Steffan-Dewenter and Kuhn^38^ and Visscher and Seeley^39^ estimated the mean foraging distance to be 1.5 and 2.3 km from the colony, corresponding to round-trip foraging flights of 3.0–4.6 km. Our control foragers flew an average of 2.1 km (acute experiment) and 3.2 km (chronic experiment). These results are similar to previous flight mill studies that used our same acute feeding procedure: control bees flew 1.8 km^30^, 2.0 km^29^ and 2.2 km^27^.

Chronic TMX exposure to 1.96–2.90 ng/bee per day reduced bee flight distance by 56% (corresponding to a 1.8 km reduction), which could lead to a decline of the overall foraging area by 79% (calculations given in the Supplementary Methods). Each 1 ng of TMX ingested per day reduced flight distance by 23% (model regression coefficient), corresponding to an estimated decline of the overall foraging area by 37% (calculations in the Supplementary Methods). TMX (0.2–2 ng/bee) can alter foragers thorax temperature up to 1 day after exposure^28^. This effect of TMX on thoracic flight muscles could impair flight because bee flight power is related to muscle temperature^40^. Tison *et al*.^34^ showed decreased honey bee foraging after chronic exposure to the neonicotinoid thiacloprid (4.5 ppm). Based upon our results, we likewise predict that bees foraging on neonicotinoid-treated fields for just one or two days will then fly more slowly and in a reduced area. This behavioural alteration should reduce the pollination service provided to plants, nectar and pollen collection for the colony, and the nutritional biodiversity of collected pollen for the colony.

Blanken *et al*.^27^ demonstrated a synergistic effect between imidacloprid (5.98 ng/mL of sucrose solution, over a 13-week period) and *Varroa* infestation on bee flight ability. They showed that foragers from colonies exposed to high levels of *Varroa* significantly decreased flight distance (−3% and −1% when respectively fed 1 M and 2 M pure sucrose solution, significant sucrose concentration effect). This effect was larger when the foragers were captured from colonies infected with *V. destructor* and chronically exposed to the neonicotinoid imidacloprid (−30% and −17% flight distance decreases when respectively fed 1 M and 2 M sucrose solution). However, there was no significant effect of imidacloprid alone on honey bee flight ability, and flight velocity was not affected even when bees were exposed to both *Varroa* and imidacloprid. We used healthy colonies that were not measurably infested with *Varroa*. Our results are therefore the first demonstration that a neonicotinoid can impair flight ability in bees that do not come from colonies heavily parasitized with *Varroa*.

Acute exposure to TMX caused excitation (hyperactivity, increased flight ability), while the chronic exposure produced depression (hypoactivity, reduced flight ability). Why did acute vs. chronic exposures lead to opposite effects? Short-term hyperactivity may lead to longer-term muscular exhaustion or energetic depletion. Neonicotinoids can impair bee energy metabolism^41^, and neonicotinoid contaminated bees have a reduced nutritional status (glycogen, lipid, and protein content) in the field^42^. The increase in sucrose consumption observed in our study may reflect bees attempting to compensate for energy deficits. For example, TMX doses of 0.2–2 ng/bee increased or decreased forager thorax temperatures depending on dose and time from exposure^28^. Cockroaches, *Blattella germanica*, showed the same response pattern to the neonicotinoid imidacloprid: individuals were hyper-responsive and hyperactive shortly after treatment, but decreased activity later on^43^. Suchail *et al*.^44^ also found differences between the acute and chronic effects of a neonicotinoid and its metabolites on bees. In their study, imidacloprid rapidly caused excitation, hyper-responsiveness and hyperactivity after intoxication. However, these symptoms gradually disappeared and, after several hours, the bees showed a decreased activity, becoming hypo-responsive and hypoactive. After a chronic exposure to the neonicotinoid imidacloprid (10 ppb), bumblebee foraging activity increased in the short-term, but was impaired in the long-term^13^. Finally, TMX degrades over time and its metabolites (including the main metabolic by-product clothianidin) could interact or differentially affect flight performance as a result of chronic exposure^5^. Clothianidin is also commonly used as a pesticide and acts on nicotinic acetylcholine receptors (nAChR). However, clothianidin targets different nAChR subtypes than TMX^5^ and possibly has dissimilar effects. The direct effects of acute and chronic exposure to neonicotinoids and similar compounds on pollinator flight ability therefore deserve further study.

## Methods

This study was conducted from September 2012 to September 2014 at University of California San Diego (UCSD), Division of Biological Sciences (La Jolla, CA, USA) with 19 healthy honey bee colonies (*A. mellifera ligustica* Spinola, 1806, 10 frames per colony) housed at an apiary in the UCSD Biology Field Station. We used standard inspection techniques^45^ to confirm that our colonies did not have measurable *Varroa* infestations.

### Preparing and flying bees

**Figure 2:**
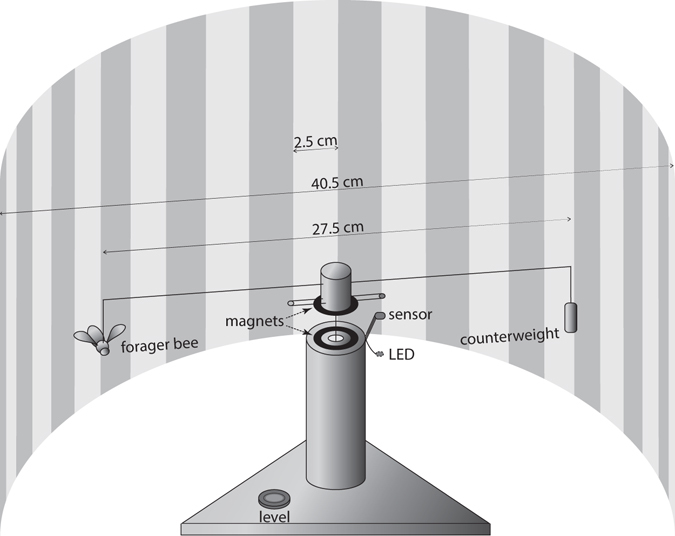
The flight mill used to test the flight ability of tethered forager bees. Foragers were attached to the wire flight mill arm through their tube harness, previously placed on top of their thorax. Once on the flight mill, bees could fly and their flight parameters were recorded by the sensor. The red LED is only triggered to light by the small triggering magnet opposite the bee, and therefore this weak red flash is not visible to the tested individual.

We tested active foragers captured upon their return to the nest. Their flight ability was tested using a modified flight mill (Fig. 2) that we built based upon the designs and software of Smith and Jones^46^. Details on the flight mill, the honey bee preparation and the flying procedure are in the Supplementary Methods.

### Pesticide doses and concentrations

Field-relevant pesticide doses and concentrations vary widely across space and time^8^. In our experiments, we used foragers fed with sucrose solution, and thus TMX levels in nectar provide the most realistic residue levels. However, honey bees can be exposed to higher concentrations of TMX in guttation droplets (100 ppm^25^), that foragers can collect from TMX seed-treated plants such as corn and oilseed rape^47^, although this may be a minor route of exposure. Even higher concentrations of TMX have been reported in bee tissue (310 ppb^23^).

We based the acute and chronic experiments and their respective analyses on the actual dose of TMX consumed by each bee. All TMX doses tested were lower than the worst case scenario thresholds, and did not increase mortality as compared to controls. The worst case scenario calculations and dose-thresholds for acute and chronic exposures were respectively defined by the European Food Safety Authority (EFSA)^48^.

In the *acute exposure* experiment, we fed the bees a single dose of TMX (1.34 ng). This dose was used by Henry *et al*.^16^ who found that it impaired forager homing ability. Using the same dose allowed us to test the hypothesis that neonicotinoids could directly impair forager flight ability. This dose is 3.7 times lower than the LD_50_ of TMX^49^ and does not significantly increase mortality as compared to controls^16^. Although 1.34 ng was subsequently criticized for not being field realistic^50^, we calculated (based upon EFSA^48^) that foragers can acutely consume up to 1.80 ng TMX/bee in 1 h of foraging for nectar (10% sugar w/w, oilseed rape^51, 52^) with a 15 ppb TMX concentration (transplant-drip application^24^). This worst case scenario considered the field-realistic amount of nectar that foragers consume in 1 h of foraging activity based upon their energy requirements. We consider 15 ppb to be a fairly high TMX concentration^24^, but even higher concentrations of TMX in nectar were found by Sanchez-Bayo and Goka^53^ (17 ppb), Dively and Kamel^24^ (19 ppb, including TMX metabolites), and Stoner and Eitzer^54^ (20 ppb, see reviews by Bonmatin *et al*.^6^ and Godfray *et al*.^7^). Transplant-drip applications are typically a short-term contamination route for bees, we therefore used this 15 ppb level to calculate the worst case acute exposure scenario: a 1 h short-term exposure to the contaminated nectar^48^. In the acute experiment, we thus tested a sublethal dose that is lower than the worst-case scenario (<1.80 ng/bee/1 h) in which bees foraged for 1 h on nectar that was contaminated by TMX after a transplant-drip application.

In the *chronic exposure* experiment, we tested a broad range of TMX daily doses (*N*_TMX daily doses_ = 46, Range_Daily doses_ 1.26–4.53 ng/bee/day, Mean_Daily doses_ = 3.1 ± 0.1 ng/bee/day) that resulted from feeding bees different concentrations of TMX. These daily doses reflected actual TMX consumption per bee cage. To identify the lowest TMX dose that significantly altered bee flight, we grouped the TMX daily doses into five bins that each spanned the same dose range (0, <1.95, <2.90, <3.71, and <4.53 ng/bee/day). EFSA estimated that foragers could consume up to 6.66 ng TMX/bee/day in a worst-case scenario^48^. This calculation considers the field-realistic amount of nectar consumed by foragers based upon their energy requirements for daily foraging activity, the sucrose content of nectar (i.e. oilseed rape, 10%, w/w^51, 52^) and the highest TMX concentration found in nectar to which bees could be chronically exposed for at least 2 days (i.e. seed treatment, 5 ppb^48^). In our experiments, foragers consumed TMX daily doses that were always lower than 6.66 ng TMX/bee/day. Furthermore, the foragers grouped in the first three bins (Fig. 1) consumed TMX daily doses that were lower than 2.94 ng/bee/day, which is the field-relevant amount of TMX that foragers can ingest when foraging on seed-treated oilseed rape producing nectar containing 20% sugar and 5 ppb TMX^48^. All tested bees remained alive throughout the experiment. Foragers have a lower sucrose requirement when incubated in cages, compared to the field, because of their reduced locomotor activity in restricted environments. This leads to lower daily sucrose consumption in cages. To test field-relevant TMX daily doses approaching a realistic worst-case scenario, foragers were provided with TMX solutions that were more concentrated (32.5 ppb or 45 ppb) than those typically found in field nectar after seed treatments. However, we focused on analyses on the field-realistic TMX daily doses consumed by our bees.

We used analytical grade TMX (CAS#153719-23-43, Sigma Aldrich 37924-100MG-R) prepared as a 25 mg/L stock solution in double-distilled H_2_0, and maintained at 4 °C inside a bottle completely wrapped in aluminium foil to avoid light degradation^6^. The solutions that we fed to bees were prepared daily by diluting the stock solution with 2.0 M glucose or 1.8 M sucrose solution for the acute and chronic experiments, respectively. These pesticide concentrations were not verified with additional chemical analyses. The rationale for using these different sugars and these concentrations is given below.

### Acute experiment

We compared the flights of bees before and after treatment. Immediately after the first flight, bees were given one of two treatments: either 10 µL of pure 2.0 M glucose solution (control treatment) or 2.0 M glucose solution with TMX (acute pesticide treatment, see above). We waited 40 min for pesticide absorption before testing their flight (similar to Henry *et al*.^16^). The density of 2.0 M glucose solution at 20 °C and 1 ATM is 1.131 kg/L^55^, and thus this dose corresponds to a solution of 118 ppb, 134 µg/L and 459 nmol/L. We used glucose because it is rapidly metabolized by bees and provides faster energy recovery than sucrose^29^.

After feeding, we placed each bee into a separate cage to prevent food exchange with other bees and maintained them in an incubator at 30 ± 1 °C, 60–70% RH, with no food for 40 min before testing their final flight. We tested 37 bees from nine colonies.

### Chronic experiment (1-day and 2-day exposures)

Bees can be chronically exposed if they continue to forage over multiple days at a food source with pesticide. We therefore tested the chronic effects of TMX. We determined how continuous exposures over different days of exposure (1 day or 2 days) would affect flight. Unlike the acute experiment, all flights occurred after pesticide treatment because we allowed bees to chronically feed from sucrose solution with pesticide.

After collection, forager bees were incubated with 1.8 M sucrose solution *ad libitum* containing either 0, 32.5 or 45.0 ppb of TMX, corresponding respectively to 0, 40, 55 µg/L and 0, 137, 190 nmol/L. The density of 1.8 M sucrose solution at 20 °C and 1 ATM is 1.230 kg/L^55^. Each day, we weighed the sucrose syringe and calculated the average sucrose and TMX consumption per cage per 24 hours and, consequently, per bee. Separately, we used 10 cages maintained in identical conditions but without bees, to measure the average mass loss (<1%) due to evaporation from the syringes. We accounted for this evaporative loss in our calculations. We tested 213 bees from 19 colonies.

### Statistical analyses

To analyse the results of the *acute* experiment, we used Repeated-Measures Analysis of Variance (ANOVA) with a REML algorithm to test the following fixed effects: treatment (control vs. pesticide-treated bees), flight period (before vs. after treatment), and their interaction on the duration (min), distance (m), mean velocity and maximum velocity (km/h) of bee flights. Colony was included as a random grouping variable. We log-transformed distance and duration and used residuals analysis to confirm that our data met parametric assumptions. Significant effects were further analysed with post-hoc Least-Square Means contrast tests.

For the *chronic* experiment, we used a Mixed Model and tested one continuous effect (TMX daily doses, *N*_*TMX daily doses*_ = 46), one fixed effect (days of exposure, 1 vs. 2), the interaction TMX daily doses × days of exposure, and colony (*N*_*Colony*_ = 19) as a random grouping variable (REML algorithm). Based on their actual TMX consumption, the bees were grouped into five bins that spanned the same dose range (Fig. 1b; 0, ≤1.95, ≤2.90, ≤3.71, ≤4.53 ng/bee/day). These ranges differ slightly from a span of 0.82 ng because we used actual consumption values to delineate the bin boundaries. We then determined the minimum dose that was significantly different from control using the Least-Square Means contrast tests and visual data inspection. To estimate the dose effect of TMX on flight parameters, we used Mixed Model estimates and assumed a linear relationship between dose and flight parameters. We log-transformed distance and duration and used residuals analysis to confirm that our data met parametric assumptions. We used the Freeman-Halton extension of the Fisher exact probability test (2 × 3, two-tailed) to test the effect of TMX treatment on the number of bees that did not fly^56, 57^. Sucrose consumption data were not normally distributed, and we therefore used a Kruskal-Wallis Rank Sums test to assess the effect of treatment on sucrose consumption and made limited post-hoc comparisons with Wilcoxon paired-sample tests.

We used JMP v10.0 statistical software and report mean ± 1 standard error (s.e.m.). We used an alpha value of 0.05. We applied stepwise model simplification, building models with all interactions and then removing them if they were not significant. The main results of the acute and chronic experiments are summarized in Table 1.

## Electronic supplementary material

Supplementary methods

Supplementary Dataset 1

Supplementary Dataset 2
