# Supplementary material for: A common neonicotinoid pesticide, thiamethoxam, impairs honey bee flight ability
Source: Sci Rep. 2017 Apr 26;7:1201. doi: 10.1038/s41598-017-01361-8 (PMC5430654; doi:10.1038/s41598-017-01361-8)
Supplement: Supplementary file 1 — Supplementary methods [file 41598_2017_1361_MOESM1_ESM.pdf]

A common neonicotinoid pesticide, thiamethoxam, impairs honey bee flight ability

Simone Tosi, Giovanni Burgio and James C. Nieh

Supplementary Methods

The flight mill

All flight mills were located in the same room which was maintained at constant light conditions (542.7 ± 1.2 Lux, mean ± s.e.m., $N = 40$, measurements made over multiple days with a digital light meter, model, LX1330B) and air temperature (25 ± 1°C, a field-realistic ambient air temperature) during flights, to avoid any influence of these parameters on bee flight ability (i.e. air temperature influence bee flight performance[1]). The flight mill allowed a tethered bee to fly, using its own power, on a light, counter-weighted arm floating on a magnetic cushion. A needle inserted into low-friction Teflon bearing kept the arm centred. With each rotation, a Hall effect magnetic sensor transmitted a voltage pulse that was recorded using LabView software v. 11.0 on a desktop PC. The time flown, distance flown and velocity were then calculated per rotation and over the entire flight with Microsoft Excel v. 14.0.

Our primary modifications to the original design[2] consisted of using a fine plastic tube to attach bees to the flight mill arm (Fig. 1) and adding a red light emitting diode (635 nm λ) on the flight mill that lit each time the Hall sensor transmitted a pulse. This allowed the operator to confirm easily that each pass of the flight arm correctly triggered the sensor. Because of the flight mill arm design (Fig. 1), this light was not visible to the honey bees because the diode lit when the bee was opposite the diode. In addition, honey bees have a poor ability to see red light[3].

To provide consistent visual feedback, the flight mill was surrounded by 40.5 cm diameter paper cylinder with laser-printed 2.5 cm wide vertical stripes alternating black and white (100% contrast, 2.5 cm spatial period), with a 6.5 cm separation between the bee and the cylinder wall.

Honey bee preparation

Foragers, identified as bees returning to the nest with corbiculae full of pollen[4,5], were individually captured in vials at hive entrances. Although the exact age of the foraging bees was not known, this method provided a more realistic sample of foraging bees. In addition, one of our goals was to compare our studies with Henry *et al.*[4], who used the same method of identifying foragers.

After collection, foragers were placed into clear plastic cages (11 x 11 x 9 cm) in groups of 10 and maintained in an incubator at 30 ± 1°C and 60-80% RH, for either 24 or 48 hours depending on

the experiment, to simulate conditions inside the nest. During incubation, bees could feed on a 1.8 M sucrose solution (pesticide-free, prepared with analytical grade sucrose and double-distilled water) provided *ad libitum* in a 5 ml syringe suspended inside the cage.

Correct harnessing is critical because bees must be securely attached with a minimal amount of thoracic adhesive to avoid impairing wing motions. First, bees were minimally chilled on ice until their motions were reduced. A wire grid (6.5 mm squares) was then lightly placed on top of each bee to restrain it during gluing. To allow a stronger attachment, the thoracic hairs were gently removed by lightly rubbing the thorax with the flat side of a wood toothpick. Next, a small quantity of contact cement (DAP® Weldwood®, Baltimore, Maryland, USA) was applied to both the end of a 1 cm-long Teflon tube (AWG22, 0.71 mm inner diameter) and to the thorax. The glue was then air-dried for 5 min before the tube was placed on top of the thorax and held steady until the adhesive was fully dry. Preliminary testing with strengthened cyanoacrylate adhesive or Pattex® contact adhesive[6] showed that the Weldwood® provided a stronger bond and required the smallest quantity of adhesive between the bee and the harness. Each bee was individually placed in a cage (11 x 11 x 9 cm) inside a dark incubator to recover from harnessing, for 40 min at nest-like conditions of 30 ± 1°C, 60-80% RH, before its flight ability was tested.

Flying bees

Using tweezers, we gently grasped the tube harness and slid it over the wire FM arm, ensuring that the bee was in the correct flight position perpendicular to the arm (Fig. 1). The slightly elastic tube walls and friction were sufficient to maintain the bee in the correct orientation. To prevent the bee from instinctively beginning to fly once its legs were no longer on the ground, we placed a small paper ball under its legs[7]. Removing the paper ball gently stimulated flight. If a bee did not start flying, we carefully removed and restored the ball once each 5 min until it began flying consistently. The bee was excluded from the experiment if she did not fly successfully within 20 minutes[5]. A flight was considered ended when the bee ceased continuous flight.

Calculations used to estimate changes in foraging area resulting from chronic exposure to thiamethoxam (TMX)

Definitions

A = foraging area

d = diameter of the foraging area

r = radius of the foraging area. We considered 1.5 km as standard foraging radius of a colony[8,9].

Formulae

$$d = 2r$$

$$A = \pi r^2$$

$$A_{\%}\ (\text{change after treatment}) = 100 - \frac{A\ (\text{treated})}{A\ (\text{control})} \times 100$$

1) Effect of 1.96-2.90 ng TMX/bee/day

r (control foragers) = 1.5 km

d (control foragers) = 2 * 1.5 km = 3.0 km

$d_{\%}$ (change after treatment) = -56%

d (change after treatment) = d (control foragers) * $d_{\%}$ (change after treatment) = 3.0 km * (-0.56) = -1.7 km

d (treated foragers) = d (control foragers) + d (change after treatment) = 3.0 km - 1.7 km = 1.3 km

r (treated foragers) = d (treated foragers) / 2 = 1.3 km / 2 = 0.7 km

A (control) = π * r (control foragers) ^{2} = π * (1.5 km)^2 = 7.1 km^2

A (treated) = π * r (treated foragers) ^{2} = π * (0.7 km)^2 = 1.5 km^2

$A_{\%}$ (change after treatment) = 100 - (1.5 km * 100 / 7.1 km) = -79%

2) Effect per each 1 ng TMX/bee/day

r (control foragers) = 1.5 km

d (control foragers) = 2 * 1.5 km = 3.0 km

$d_{\%}$ (change after treatment, based on model regression coefficient) = -23%

d (change after treatment) = d (control foragers) * $d_{\%}$ (change after treatment) = 3.0 km * (-0.23) = -0.7 km

d (treated foragers) = d (control foragers) + d (change after treatment) = 3.0 km - 0.7 km = 2.3 km

r (treated foragers) = d (treated foragers) / 2 = 2.3 km / 2 = 1.2 km

A (control) = π * r (control foragers) ^{2} = π * (1.5 km)^2 = 7.1 km^2

A (treatment) = π * r (treated foragers) ^{2} = π * (1.2 km)^2 = 4.5 km^2

$A_{\%}$ (change after treatment) = 100 - (4.5 km * 100 / 7.1 km) = -37%

References cited in Supplementary Methods

1. Hrassnigg, N. & Crailsheim, K. Metabolic rates and metabolic power of honeybees in tethered flight related to temperature and drag (Hymenoptera: Apidae). *Entomol. Gen.* **24,** 23–30 (1999).
2. Smith, T. & Jones, V. P. The Flight Mill http://entomology.tfrec.wsu.edu/VPJ_Lab/Flight-

Mill. (2012).

3. Briscoe, A. D. & Chittka, L. The evolution of color vision in insects. *Annu. Rev. Entomol.* **46,** 471–510 (2001).
4. Henry, M. *et al.* A common pesticide decreases foraging success and survival in honey bees. *Science (80-.).* **336,** 348–350 (2012).
5. Blanken, L. J., Langevelde, F. Van & Dooremalen, C. Van. Interaction between Varroa destructor and imidacloprid reduces flight capacity of honeybees. *Proc. R. Soc. B* **282,** 20151738 (2015).
6. Scheiner, R. *et al.* Standard methods for behavioural studies of Apis mellifera. *J. Apic. Res.* **52,** 1–58 (2013).
7. Gmeinbauer, R. & Crailsheim, K. Glucose utilization during flight of honeybee (Apis mellifera) workers, drones and queens. *J. Insect Physiol.* **39,** 959–967 (1993).
8. Steffan-Dewenter, I. & Kuhn, A. Honeybee foraging in differentially structured landscapes. *Proc. Biol. Sci.* **270,** 569–575 (2003).
9. Visscher, P. K. & Seeley, T. D. Foraging strategy of honeybee colonies in a temperate deciduous forest. *Ecol. Soc. Am.* **63,** 1790–1801 (1982).
